# Histone lactylation in macrophage biology and disease: from plasticity regulation to therapeutic implications

**DOI:** 10.1016/j.ebiom.2024.105502

**Published:** 2024-12-10

**Authors:** Chuncha Bao, Qing Ma, Xihong Ying, Fengsheng Wang, Yue Hou, Dun Wang, Linsen Zhu, Jiapeng Huang, Chengqi He

**Affiliations:** a Department of Rehabilitation Medicine, West China Hospital, Sichuan University, Chengdu, Sichuan 610041, PR China; b Key Laboratory of Rehabilitation Medicine, West China Hospital, Sichuan University, Chengdu, PR China; c General Practice Ward/International Medical Center Ward, General Practice Medical Center, West China Hospital, Sichuan University /West China School of Nursing, Sichuan University, Chengdu, Sichuan 610041, PR China; d Clinical Medical College of Acupuncture-Moxibustion and Rehabilitation, Guangzhou University of Chinese Medicine, Guangzhou, Guangdong 510006, PR China; e State Key Laboratory of NBC Protection for Civilian, Beijing 102205, PR China

**Keywords:** Macrophages, Lactate, Histone lactylation, Epigenetic modifications, Plasticity, Diseases

## Abstract

Epigenetic modifications have been identified as critical molecular determinants influencing macrophage plasticity and heterogeneity. Among these, histone lactylation is a recently discovered epigenetic modification. Research examining the effects of histone lactylation on macrophage activation and polarization has grown substantially in recent years. Evidence increasingly suggests that lactate-mediated changes in histone lactylation levels within macrophages can modulate gene transcription, thereby contributing to the pathogenesis of various diseases. This review provides a comprehensive analysis of the role of histone lactylation in macrophage activation, exploring its discovery, effects, and association with macrophage diversity and phenotypic variability. Moreover, it highlights the impact of alterations in macrophage histone lactylation in diverse pathological contexts, such as inflammation, tumorigenesis, neurological disorders, and other complex conditions, and demonstrates the therapeutic potential of drugs targeting these epigenetic modifications. This mechanistic understanding provides insights into the underlying disease mechanisms and opens new avenues for therapeutic intervention.

## Introduction

Macrophages reside throughout the body as tissue-resident cells or as differentiated products of circulating monocytes.^1^ They play a critical role in maintaining homeostasis, responding dynamically to various internal and external cues, including microenvironmental shifts, hypoxia, inflammation, immune challenges, and tumor presence.^2^, ^3^, ^4^, ^5^ All macrophages are highly plastic, adjusting their phenotypes in response to environmental changes.^6^ Studies indicate that macrophage phenotypes exist on a spectrum, rather than as fixed states, reflecting variations in cytokine profiles and functional characteristics.^7^^,^^8^ These phenotypic shifts are closely tied to epigenetic regulation. Epigenetics refers to the modulation of gene expression and modification processes without altering the underlying DNA sequences, facilitating direct interactions between genes and the environment.^9^, ^10^, ^11^ One significant epigenetic alteration is the post-translational modification (PTM) of chromatin, which plays a crucial role in regulating cellular characteristics.^12^ Current chromatin modification methods include methylation, acetylation, ubiquitination, glycosylation, phosphorylation, and lactylation.^13^ Recently, histone lactylation has emerged as a key epigenetic modification, significantly influencing protein functional diversity.^14^^,^^15^ Zhao et al. analyzed core histones in the human breast cancer cell line MCF-7 using HPLC-MS/MS, revealing that L-lactic acid coenzyme A (the activated form of L-lactic acid) can dynamically promote the lactylation of histone lysine sites.^16^^,^^17^ Additionally, they identified 26 and 16 lactylation sites in the core histones from HeLa cells and bone marrow-derived macrophages (BMDMs), respectively.^17^ These findings mark a pivotal advancement in lactate research, opening new avenues for understanding histone PTMs and offering novel insights into immune regulation and cellular microenvironment maintenance.

Similar to classical histone modifications such as acetylation and methylation, lactylation is integral to various physiological and pathological processes by influencing chromatin structure and gene expression. Recent studies indicate that histone lactylation not only participates in metabolic reprogramming in tumor cells but also plays essential regulatory roles in immune cells, including macrophages and T cells.^18^^,^^19^ Notably, under metabolic stress, histone lactylation levels significantly increase, suggesting its potential as a critical link between cellular metabolic states and epigenetic regulation. However, the dynamic patterns of histone lactylation and its regulatory mechanisms on macrophage functions remain poorly understood, hindering comprehensive insights into this modification's role in disease progression. This review presents the first systematic analysis of macrophage histone lactylation's regulatory roles across major diseases, including neurological disorders, inflammatory diseases, and cancer. By synthesizing recent advances, we elucidate the molecular connections between macrophage metabolic reprogramming and histone lactylation, along with their regulatory mechanisms in macrophage polarization and inflammatory responses. Furthermore, we summarize the latest developments in targeting macrophage histone lactylation for therapeutic interventions. This review not only addresses a critical knowledge gap but also establishes a theoretical foundation for developing innovative therapeutic strategies targeting histone lactylation.

### From lactate metabolism to histone lactylation modification

Lactate, the final product of glycolysis, serves as a metabolic fuel in tissues such as the brain, heart, and skeletal muscle.^20^ It also acts as a signaling molecule in various physiological and pathological processes, including immune responses, inflammation, energy homeostasis, tumor growth, and the tumor microenvironment (TME).^21^^,^^22^ Lactate is transported within and between cells primarily via monocarboxylate transporters (MCTs) that facilitate its movement across the plasma membrane. Interestingly, this transport is synergistically influenced by pH, lactate concentration gradients, and cellular redox reactions.^23^^,^^24^ More than 10 MCT types have been identified, each with a specific transport direction.^25^ When intracellular lactate concentration and pH decrease, the expression level of MCT1 protein on the cell membrane surface can be upregulated, thereby enhancing the uptake of intracellular lactate. Conversely, when intracellular lactate concentration and pH value increase, MCT4 protein expression can rise, promoting lactate efflux.^25^^,^^26^ The intracellular lactate level affects histone lactylation in a dose-dependent manner.^27^ Glycolytic inhibitors reduce histone lactylation by lowering lactate levels, while supplementation with glucose or mitochondrial inhibitors to elevate lactate levels increases histone lactylation.^17^^,^^28^^,^^29^

**Fig. 1:**
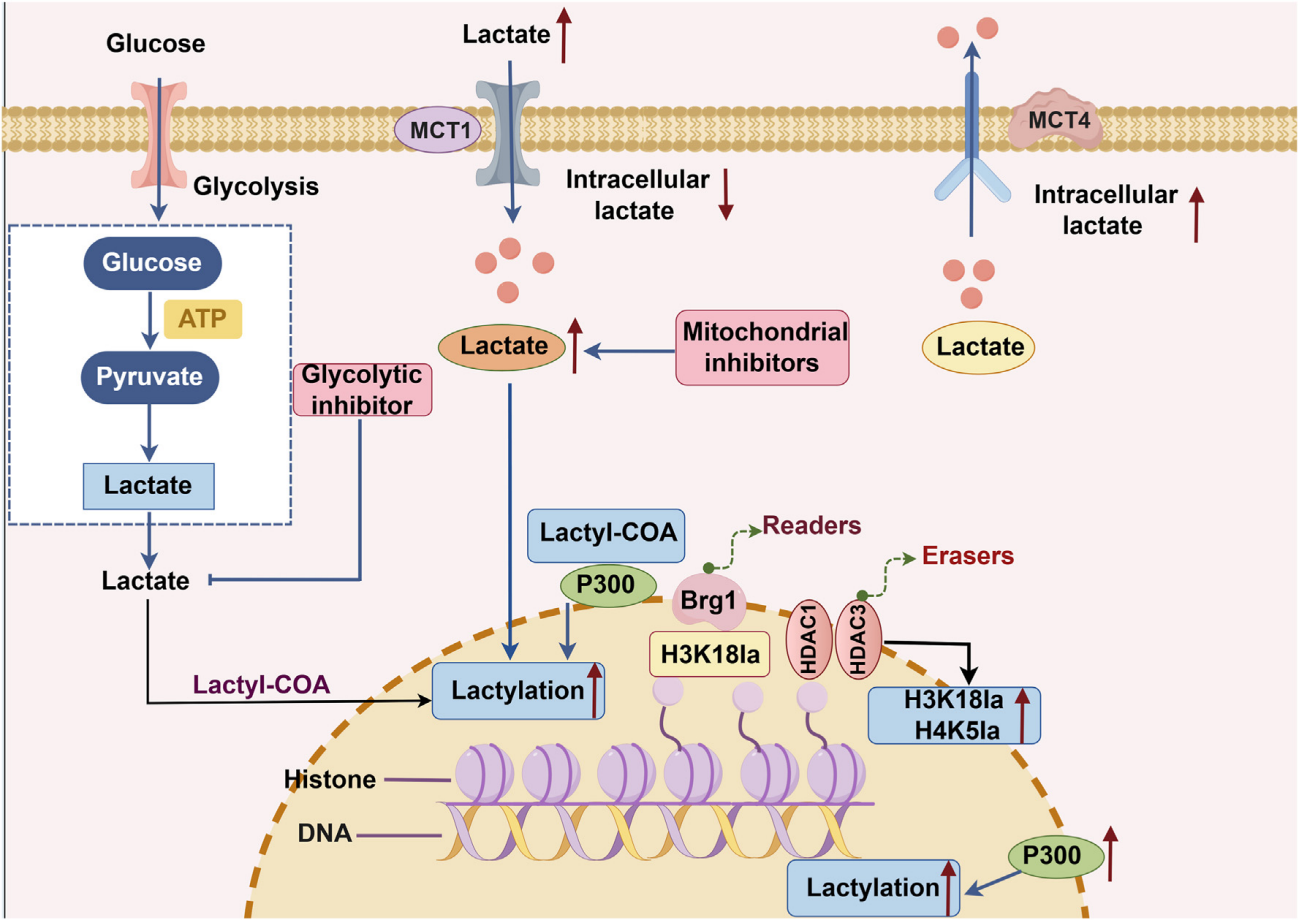
Metabolites and modifying enzymes that affect histone lactate levels. Lactate, produced during glycolysis or transported by the MCT1 transporter protein, significantly influences histone lactylation levels. Glycolytic inhibitors decrease lactylation by lowering lactate levels, while mitochondrial inhibitors that elevate lactate concentrations enhance lactylation. Histone lactate levels are modulated by readers such as Brg1 and regulated by erasers such as HDAC1 and HDAC3. Furthermore, p300 catalyzes histone lactate modifications; alterations in p300 expression can increase or decrease histone lactate levels.

Histone lactylation, a novel post-translational modification (PTM) mediated by lactate, was first identified in 2019.^17^ Like other PTMs, lactylation modifies histone lysine residues, altering chromatin structure and subsequently affecting gene regulation.^30^ Similar to methylation and acetylation, histone lactylation levels are governed by “readers” (proteins that recognize lactylation to perform specific functions), “writers” (specific lactylases), and “erasers” (de-lactylases).^20^ Despite its significance, there are relatively few studies elucidating the regulatory mechanisms of histone lactylation by these enzymes. Zhang et al. identified five effective lactate CoA transferases in cells using liquid chromatography-mass spectrometry (LC-MS), which play crucial roles in regulating physiological and pathological conditions.^31^ The histone-modifying enzyme p300 functions as a lactyltransferase, catalyzing histone lactylation.^31^ Research indicates that overexpression or inhibition of p300 in HEK293T cells leads to increased or decreased histone lactylation levels, suggesting its catalytic role in lactate modification within these cells.^31^ Among that, lactyl coenzyme A (lactyl-CoA) serves as a substrate for lysine lactylation catalyzed by p300.^32^ Histone deacetylases 1 (HDAC1) and 3 (HDAC3) exhibit site-specific de-lactylase activity in cells, reducing lactylation at H3K18 and H4K5. Notably, knockdown of HDAC3 significantly increases H4K5 lactylation, indicating that HDAC1 and HDAC3 effectively function as “erasers” of histone lactylation modifications.^33^ Proteins with the ability to recognize and bind specific epigenetic modifications are commonly referred to as “readers”.^34^ Recent research has revealed the specific recruitment of the bromodomain-containing protein Brg1 during reprogramming, as demonstrated through proteomic analysis of H3K18 lactylation immunoprecipitation experiments. The interaction between Brg1 and H3K18 lactylation promotes Brg1 accumulation at the promoters of MET-related genes, supporting its role as a reader of histone lactylation and enhancing our understanding of the reading enzymes involved in this modification^34^ (Fig. 1).

### Diversity and phenotype of macrophages

Macrophages, myeloid mononuclear cells with phagocytic capabilities, are ubiquitous in nearly all tissues,^35^ including circulating monocytes, resident macrophages, and dendritic cells.^36^ Tissue-resident macrophages are present in most tissues and possess both general and tissue-specific functions. Examples include osteoclasts in bone, microglial cells in neural tissue, Kupffer cells in the liver, tumor-associated macrophages (TAMs), and alveolar macrophages in the lungs.^37^, ^38^, ^39^ Some tissue-resident macrophages are derived from peripheral blood monocytes. For instance, under stimulation by macrophage colony-stimulating factor (M-CSF), hematopoietic stem cells in the bone marrow generate the monocyte/macrophage lineage, which further differentiates into osteoclasts.^40^^,^^41^ In neurological disorders, bone marrow-derived macrophages can compensate for dysfunctional microglia, facilitating the clearance of toxic substances and cellular debris. This involvement extends to various cerebrovascular diseases and neurodegenerative diseases.^42^ Under normal conditions, monocyte-derived macrophages predominantly circulate in peripheral blood. Upon exposure to external stimuli, these monocytes migrate into specific tissues, where they differentiate into macrophages, thus regulating inflammatory and immune responses.^43^^,^^44^ Additionally, circulating monocytes can be recruited to mucosal tissues and inflamed sites, differentiating into monocyte-derived macrophages (mo-Mac) or monocyte-derived dendritic cells (mo-DC).^45^^,^^46^ In inflammatory diseases and injured tissues, monocyte recruitment can be substantial, prompting local differentiation into infiltrating or inflammatory macrophages.^47^ These cells modulate inflammatory responses through cellular polarization and metabolic reprogramming, further contributing to tissue damage and functional repair.^48^^,^^49^

Macrophages exhibit significant heterogeneity and polarization. Under pathological conditions induced by various stimuli, such as microbes, tissue microenvironments, and cytokine signaling, activated macrophages polarize into distinct phenotypes, primarily M1 and M2, each performing different roles and functions.^50^ M1 macrophages, also referred to as classically activated macrophages, are induced by toll-like receptor (TLR) ligands (such as LPS) and Th1 cytokines (e.g., interferon gamma [IFN-γ]). They secrete pro-inflammatory cytokines, including tumor necrosis factor α (TNF-α), IL-1β, IL-6, and IL-23.^47^^,^^51^^,^^52^ M1 macrophages are effective in eliminating bacteria, viruses, and tumor cells, as well as phagocytizing bacterial debris and dead cell remnants.^53^^,^^54^ Thus, M1 macrophages play a crucial role in infection prevention and exert anti-tumor and immune functions, contributing to homeostasis in the human body.^54^ In contrast, M2 macrophages, known as anti-inflammatory macrophages, are activated by IL-4 and IL-13, leading to the secretion of anti-inflammatory factors such as IL-10, transforming growth factor-beta (TGF-β), and arginase 1 (Arg1).^55^, ^56^, ^57^

M2 macrophages can be further classified into distinct subtypes (M2a, M2b, M2c, and M2d), each characterized by specific stimuli and unique functional profiles.^58^, ^59^, ^60^ M2a macrophages, also termed wound healing macrophages, are linked to anti-inflammatory effects. Under M2a polarizing conditions (primarily IL-4/IL-13 stimulation), they exhibit increased expression of markers like CD206 and Arg1, alongside heightened secretion of CCL17, CCL22, and IL-10.^61^ M2b macrophages, recognized as regulatory macrophages, possess both pro-inflammatory and anti-inflammatory functions. When stimulated by a combination of lipopolysaccharide (LPS) and immune complexes (IC), M2b macrophages express TNF superfamily member 14 (TNFSF14) at elevated levels.^61^ Furthermore, M2b cells can secrete pro-inflammatory cytokines such as IL-1β, IL-6, and TNF-α, in addition to the anti-inflammatory cytokine IL-10.^62^ M2c macrophages, referred to as acquired deactivated macrophages, exhibit strong anti-inflammatory activity by releasing substantial amounts of IL-10.^63^ They enhance the clearance of apoptotic cells, particularly under steroid stimulation.^64^ M2d macrophages, known as tumor-associated macrophages (TAMs), upregulate IL-6, IL-10, TGF-β, VEGF, and PD-L1 expression while depleting arginine to suppress T cell anti-tumor functions. This mechanism promotes tumor growth and progression, including invasion, immune evasion, and treatment resistance.^65^, ^66^, ^67^, ^68^

## Role of histone lactylation changes in macrophages in various diseases

### Central nervous system diseases

Microglia, the resident macrophages of the central nervous system (CNS), constitute 5%–12% of all CNS cells. They primarily originate from erythromyeloid progenitors in the yolk sac.^69^^,^^70^ Once in the brain, microglia sustain a dynamic equilibrium through slow, localized proliferation stimulated by cytokines (IL-34 and colony-stimulating factor (CSF)-1) and transcription factors (PU.1 and interferon regulatory factor 8).^71^, ^72^, ^73^ Under pathological conditions, microglia respond rapidly to subtle microenvironmental changes, playing key roles in pathogen clearance, neuronal and glial cell maintenance, and immune surveillance.^74^ Numerous studies have linked microglial dysfunction to the progression of various brain diseases, including Alzheimer's disease (AD),^75^ Parkinson's disease (PD),^76^ multiple sclerosis (MS),^77^ neuroinflammation,^78^ and brain tumors.^79^ However, the underlying mechanisms driving microglial dysfunction remain to be fully elucidated. Recent advances in single-cell and nucleus sequencing technologies have identified distinct microglial phenotypes associated with disease progression.^80^^,^^81^ Epigenetic regulation is pivotal in modulating microglial activation and phenotypes. Notably, several mechanisms, such as histone lactylation, have been shown to correlate closely with microglial activation states.^80^

**Fig. 2:**
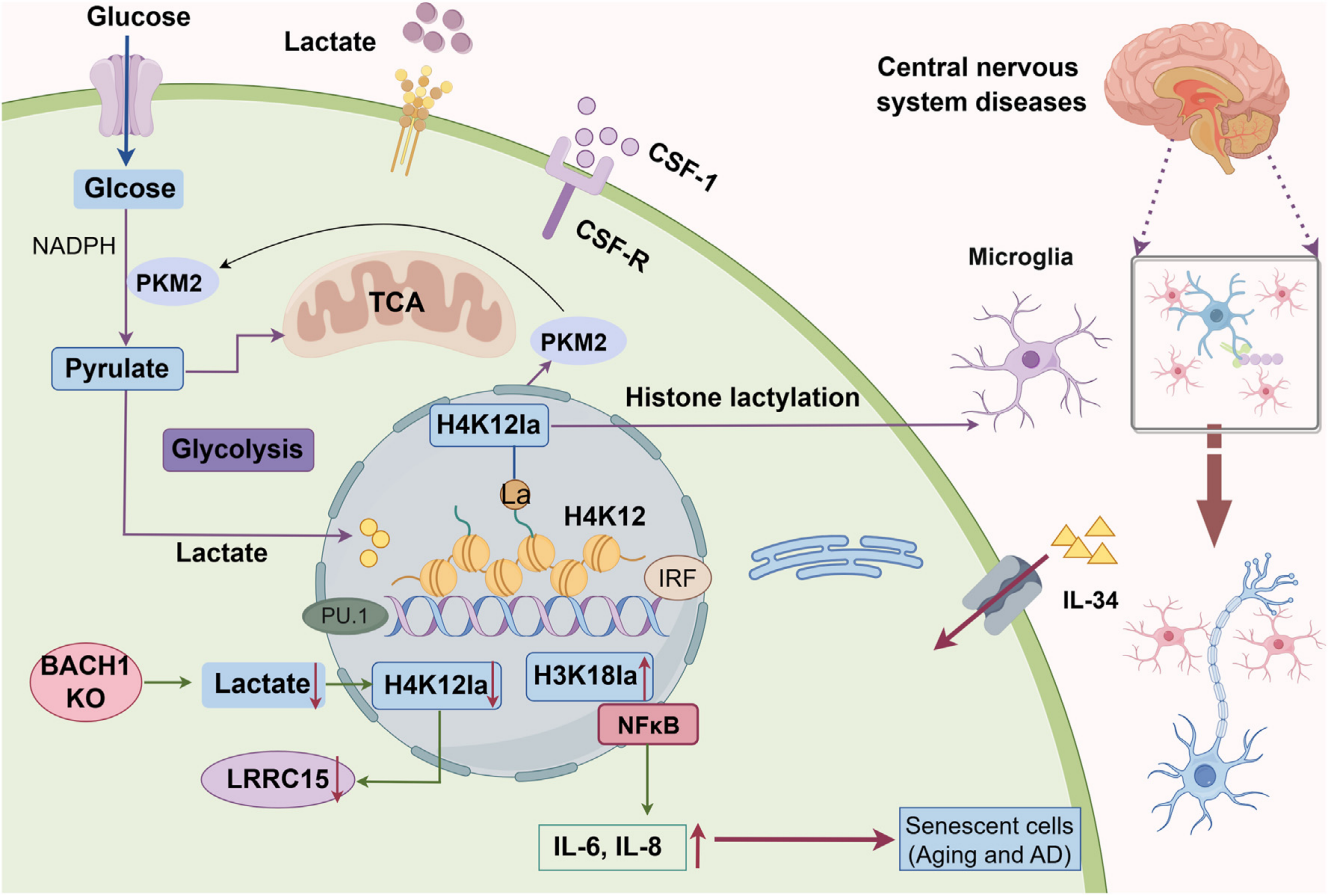
Role of histone lactylation changes in macrophages in central nervous system diseases. Microglia, the resident macrophages of the CNS, are closely linked to the development of various brain diseases. Histone lactylation modifications play a crucial role in modulating microglial activation and phenotypes. Increased lactate levels or PKM2 during glycolysis can upregulate NF-κB, IL-6, and IL-8 expression in microglia by promoting histone lactylation. Conversely, disruption of Bach1 reduces H4K12la enrichment at the Lrrc15 promoter.

Cellular metabolic reprogramming and histone lactylation modifications engage in reciprocal interactions; metabolic changes in microglia can lead to the accumulation of metabolites that influence histone lactylation.^82^ A recent study demonstrated that microglia from an AD mouse model (5XFAD) exhibit increased lactate production and histone lactylation levels. Analysis using whole-genome targets and the CUT&Tag technique identified PKM2 as an H4K12Ia-regulated gene in AD microglia. This finding indicates that the glycolysis/H4K12Ia/PKM2 positive feedback loop exacerbates microglial activation and dysfunction in AD.^82^ Moreover, specific knockout of PKM2 in microglia of AD models disrupts this signaling loop, inhibiting microglial activation and alleviating Aβ pathology in 5XFAD mice.^82^ In aging microglia (BV2 and HMC3 cells) and senescent glial cells from AD mice, significantly elevated levels of H3K18la have been observed. Combined analysis of ChIP-qPCR and RNA-seq data further revealed that lactate-induced elevation of H3K18la directly activates NF-κB signaling by enhancing binding to the NF-κB1 (p50) promoter region, which in turn upregulates senescence-associated secretory phenotype (SASP) components IL-6 and IL-8, thereby influencing brain aging and phenotypes of Alzheimer's disease.^83^^,^^84^ Similarly, in a spinal cord injury (SCI) model, Hu et al. found that elevated endogenous or exogenous lactate levels, induced by ischemia and hypoxia, promote microglial scar formation and enhance motor function recovery by increasing H4K12 lactylation levels (H4K12la).^85^ Histone lactylation is also vital for normal brain development and homeostasis. In a genome-wide analysis, Dai et al. demonstrated that histone lactylation is widespread in newborn brain tissue, with significant alterations in H3K18 lactylation during neural development.^32^ The metabolic homeostasis of microglia and histone lactylation are critical for proper mouse brain development. Disruptions in microglial metabolism can reduce histone lactylation enrichment at the Lrrc15 gene promoter. Specifically, in Bach1 conditional knockout (cKO) microglia, H4K12la enrichment at the Lrrc15 promoter is diminished, which affects the JAK/STAT signaling pathway and subsequently influences astrocyte genesis.^83^ These findings indicate that histone lactylation levels can become dysregulated in various central nervous system (CNS) diseases due to aging, metabolic dysregulation, and ischemia/hypoxia. Changes in cellular metabolic states, particularly metabolic reprogramming and lactate accumulation, serve as a primary driver for dysregulation in histone lactylation. Such metabolic-epigenetic changes can initiate pathological cascades by modulating key signaling pathways and gene expression programs, ultimately contributing to disease progression. Additionally, Han et al. showed that exercise training in a mouse model of Alzheimer’s disease (AD) can alleviate cognitive dysfunction and neuroinflammation via histone H3 lactylation in microglia. This phenomenon may be due to exercise-induced increases in brain lactate levels, which act as an “accelerator” for the microglial “lactate timer.” This lactate modification inhibits excessive microglial activation in AD-like mice and boosts the expression of microglial repair genes, thus maintaining brain homeostasis^86^ (Fig. 2).

In summary, these studies underscore the diverse role of histone lactylation in regulating microglial function, from neurodevelopment to disease intervention. In CNS diseases such as AD and SCI, alterations in cellular metabolic states—particularly metabolic reprogramming and lactate accumulation—are primary drivers of dysregulated histone lactylation in microglia. These metabolic-epigenetic changes influence key signaling pathways and gene expression, initiating pathological cascades that ultimately promote disease progression. Despite this progress, the complexity of neurological diseases leaves many aspects of histone lactylation-mediated regulation in microglia, along with associated molecular mechanisms in various CNS diseases, yet to be explored.

### Tumor-associated macrophages (TAMs)

**Fig. 3:**
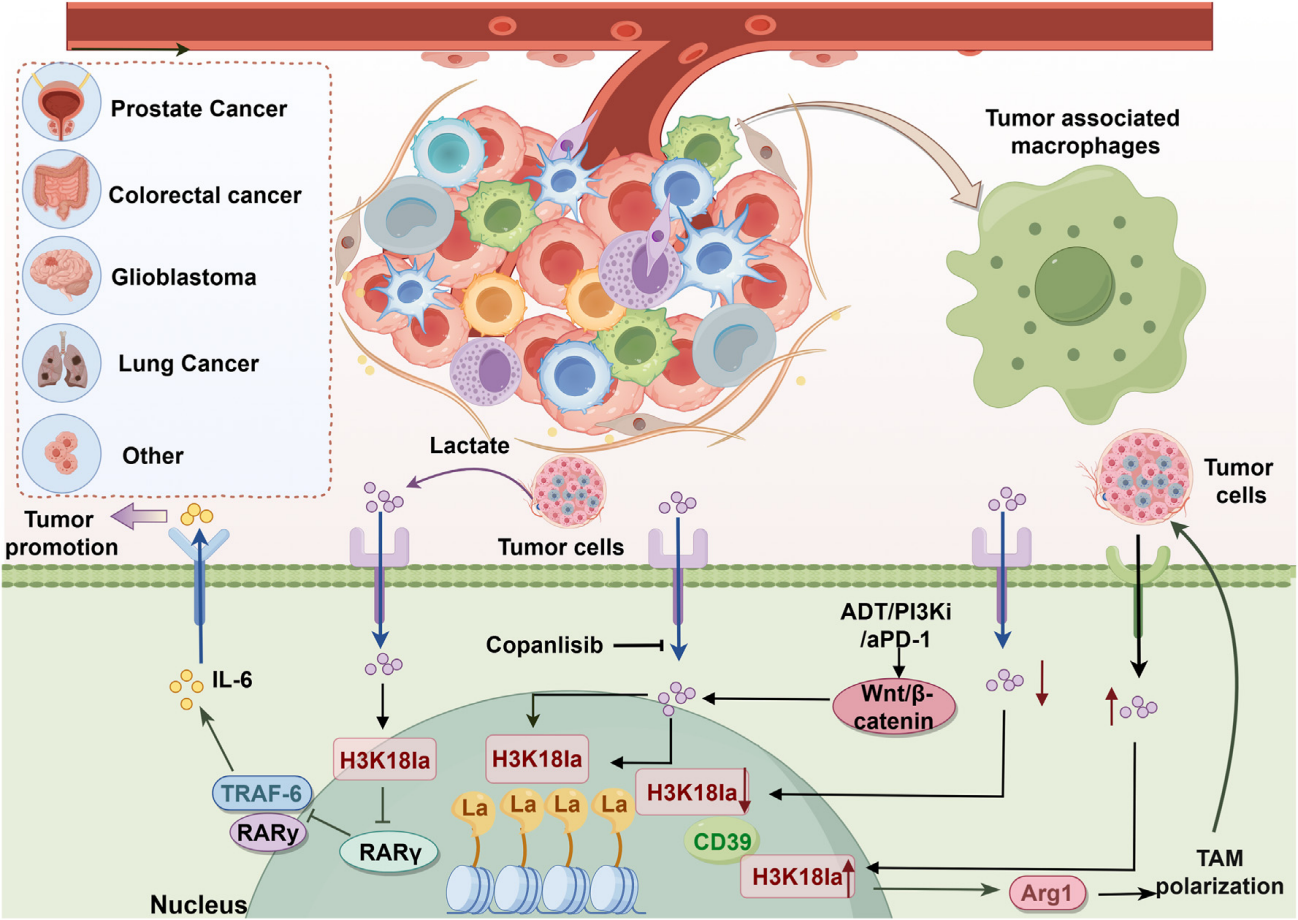
Role of histone lactylation changes in tumor-associated macrophages. During tumor development, macrophages exhibit significant phenotypic plasticity. They utilize lactate, a key metabolite in the tumor microenvironment (TME), to modify their phenotype through histone lactate modifications, influencing tumor progression. In colorectal cancer (CRC), tumor-derived lactate inhibits the transcription of the RARγ gene in macrophages via H3K18 lactylation, stimulating the TRAF6-IL-6-STAT3 signaling pathway to support tumor growth. In prostate cancer, PI3K-inhibited tumor cells suppress histone lactylation in tumor-associated macrophages (TAMs), promoting their anti-cancer phagocytic activation. This activation is enhanced by androgen deprivation therapy (ADT) combined with anti-PD-1 treatment but is countered by the feedback activation of the Wnt/β-catenin pathway. Similarly, in glioblastoma (GBM), lactate induces H3K18 lactylation in macrophages, upregulating the expression of CD39.

The tumor microenvironment (TME) represents the ecological niche surrounding the tumor, significantly influencing tumorigenesis, progression, and metastasis.^87^ Composed of various cells with unique metabolic characteristics, tumor-associated macrophages (TAMs) are a major component of the TME and are often linked to poor prognosis and therapeutic resistance in various tumors.^88^ Macrophages can differentiate into two distinct polarization states: classically activated “M1” macrophages, which are pro-inflammatory, and alternatively activated “M2” macrophages, which are anti-inflammatory.^89^^,^^90^ Tumor-associated macrophages (TAMs) represent a heterogeneous population of phagocytic cells with unclear origins.^91^ During tumor development, macrophages exhibit considerable phenotypic plasticity, displaying a spectrum of activation states that range from M1 to M2-like characteristics, rather than conforming to a strict binary classification. This phenotypic diversity underscores the significance of understanding macrophage polarization dynamics for the development of effective immunotherapy strategies.^92^^,^^93^ Furthermore, the metabolic profile of macrophages is influenced by the tumor microenvironment (TME), which plays a crucial role in the reprogramming of TAMs^94^(Fig. 3).

Metabolic reprogramming is a hallmark feature of cancer, enabling tumor cells to sustain growth and proliferation within a nutrient-deprived TME.^95^ During rapid tumor expansion, abnormal angiogenesis results in insufficient oxygen supply, leading to hypoxic stress. This condition drives tumor cells to adapt metabolically, predominantly relying on glycolysis for energy production, even under aerobic conditions—a phenomenon known as the Warburg effect. One consequence of this metabolic reprogramming is the substantial accumulation of lactate in the TME.^96^^,^^97^ Elevated lactate levels promote tumor progression through various mechanisms; for instance, lactate accumulation creates an acidic environment that suppresses immune cell function and facilitates tumor immune escape. This acidic microenvironment also enhances tumor cell invasion and metastatic potential.^98^ Importantly, studies have shown that lactate can induce histone lactylation, a unique epigenetic modification. In TAMs, lactate-induced histone lactylation alters their epigenetic landscape, driving polarization toward a pro-tumoral phenotype.^97^ This reprogramming enhances the immunosuppressive functions of TAMs while supporting tumor growth through mechanisms such as the secretion of pro-angiogenic factors, thereby establishing a vicious cycle that promotes tumor progression.

Macrophages can utilize lactate, a key metabolite in the TME, to modify their phenotype via histone lactylation, subsequently influencing tumor progression.^99^ Zhang et al. first identified histone lactylation in tumor cells and later detected this modification in TAMs isolated from mouse melanoma and lung cancer tissues. They found that levels of histone lactylation positively correlated with Arg1 expression and the tumorigenic properties of M2 macrophages.^17^ Notably, tumor cells generate abundant lactate both under hypoxic conditions and during aerobic glycolysis dominated by the Warburg effect, providing ample substrate for histone lactylation. Pro-tumoral TAMs display distinct molecular characteristics across various cancer types, including colorectal cancer (CRC).^100^ In CRC, downregulation of RARγ in macrophages correlates with poor prognosis.^99^ Specifically, tumor-derived lactate inhibits RARγ gene transcription in macrophages through H3K18la, activating TRAF6-IL-6-STAT3 signaling to support tumor growth.^99^ This study highlights the role of histone lactylation-driven RARγ downregulation in promoting pro-inflammatory and pro-tumor phenotypes in macrophages, revealing a novel mechanism by which histone lactylation in TAMs advances tumor progression.^99^ Additionally, another study indicated that in CRC, the m6A methyltransferase METTL3 and histone lactylation work together to enhance Kcnk6 stability in a YTHDF2-dependent manner, promoting macrophage inflammation-associated carcinogenesis. This research suggests that histone lactylation may interact with other epigenetic modifications to collectively drive tumor progression.^101^ Further studies have reported that in prostate cancer, tumor-derived lactate induces histone lactylation in TAMs, reprogramming their function. Inhibition of the PI3K pathway reduces tumor lactate production, consequently decreasing TAM histone lactylation and promoting anti-tumor phagocytic activity. However, this therapeutic effect is diminished by compensatory Wnt/β-catenin activation, highlighting the complex regulatory network surrounding histone lactylation in tumors.^102^

Recent studies underscore the critical role of histone lactylation in macrophages and its contribution to tumor immune evasion. Ectonucleotidases CD39 and CD73, key enzymes in adenosine metabolism, have emerged as crucial players in establishing an immunosuppressive tumor microenvironment (TME). In glioblastoma (GBM), tumor-derived lactate induces H3K18 lactylation, leading to increased expression of CD39, CD73, and CCR8. These changes disrupt the balance between regulatory T cells (Treg) and T helper 17 (Th17) cells, elucidating how tumor-derived lactate promotes immunosuppression in the TME through epigenetic regulation via histone lactylation.^103^ Additionally, a recent study highlights a novel mechanism of histone lactylation in immune evasion within tumor-associated macrophages (TAMs). This research shows that SRSF10 enhances RNA stability by binding to the 3' UTR of MYB, upregulating the expression of key glycolytic enzymes (GLUT1, HK1, LDHA), and ultimately increasing intracellular and extracellular lactate levels.^19^ In turn, the accumulated lactate induces H3K18 lactylation, promoting SRSF10 expression and establishing a positive feedback loop between SRSF10, glycolysis, and H3K18 lactylation. Moreover, lactate secreted by tumor cells is taken up by TAMs, driving M2 macrophage polarization and constructing an immunosuppressive microenvironment by inhibiting CD8+ T cell infiltration and IFN-γ secretion.^19^ Conversely, another study found that endogenous lactate in lung tumor cells is not essential for TAM polarization and tumor progression. Instead, extracellular lactate induces TAM polarization through HIF-1α stabilization and histone lactylation, which are crucial for promoting TAM polarization and tumor growth.^104^ Hypoxia, a critical factor contributing to poor tumor prognosis, has been shown to promote macrophage polarization toward an M2 phenotype, further supporting tumor progression. However, the underlying mechanisms remain to be fully elucidated.^105^ A recent study revealed that hypoxia fosters lactate accumulation in glioma cells, which is subsequently taken up by macrophages, leading to M2 polarization via the MCT-1/H3K18 lactylation/TNFSF9 signaling pathway, ultimately promoting glioma progression.^105^ This research provides new insights into the connection between glioma energy metabolism and macrophage histone lactylation.

In summary, tumor cells produce large amounts of lactate due to hypoxia, increased glycolytic activity, gene regulation, and increased glucose uptake. Increased intracellular lactate levels which are subsequently transported into the TME via monocarboxylate transporters (MCT1 or MCT4), creating an acidic environment that affects nearby macrophages. When taken up by TAMs, these tumor-derived lactates induce histone lactylation modifications that promote macrophage polarization toward the pro-tumoral M2 phenotype. This epigenetic modification further regulates downstream genes, ultimately constructing an immunological microenvironment that facilitates tumor progression. It can be inferred that histone lactylation in macrophages (key cells in the immune microenvironment), may act as a promoting or supporting factor in macrophage phenotypic transitions (such as M1 to M2) and exerts a unique influence on immune regulation within the tumor microenvironment. This lactate-mediated intercellular transport not only reflects metabolic interactions between tumor and immune cells but also contributes to the immunosuppressive characteristics of the TME. However, future research is needed to determine the causes of changes in histone lactylation levels within macrophages and elucidate the molecular mechanisms of downstream regulation.

### Inflammatory diseases

Macrophages, as highly plastic cells of the innate immune system, play a crucial role in promoting and modulating inflammatory responses through diverse functions.^106^^,^^107^ During inflammatory diseases, they express various pattern recognition receptors (PRRs) that allow them to sense their microenvironment and adjust their behavior and phenotype, thereby facilitating appropriate inflammatory responses.^108^ In the tissue repair process, macrophages undergo metabolic reprogramming to fulfill specific functions at different stages.^109^ Recent studies have identified a unique epigenetic modification in inflammatory macrophages, known as histone lactylation, which is dependent on aerobic glycolysis following classical M1 polarization. This process results in elevated lactate levels that promote histone lactylation and selectively upregulate repair-associated genes. Although the precise molecular mechanisms remain under investigation, a pivotal study demonstrated that BCAP-deficient macrophages exhibited significantly reduced expression of repair-associated genes, including Arg1 and Klf4. Notably, when treated with Nala or exposed to elevated histone lactylation, BCAP−/− bone marrow-derived macrophages (BMDMs) restored Arg1 expression to levels comparable to wild-type BMDMs within 12 h, along with a significant increase in Klf4 expression^110^ Similarly, another study further indicated that elevated histone lactylation correlates with the expression of the repair gene arginase-1 (Arg1) in M2 macrophages, coinciding with Arg1-dependent metabolic rewiring in inflammatory environments.^111^ Recent investigations have uncovered a novel link between mitochondrial dynamics and histone lactylation during inflammation resolution. M2-polarized macrophages display a more fragmented mitochondrial network than M1 macrophages, with the absence of mitochondrial fusion resulting in increased baseline Arg1 expression. Mechanistically, cells with fragmented mitochondria accumulate higher lactate levels, leading to enhanced histone lactylation and promoting the transition of macrophages toward an anti-inflammatory phenotype.^112^ Together, these studies illuminate a complex regulatory network in which metabolic state, mitochondrial dynamics, and histone lactylation converge to orchestrate macrophage phenotypic transitions, particularly during inflammation resolution.

**Fig. 4:**
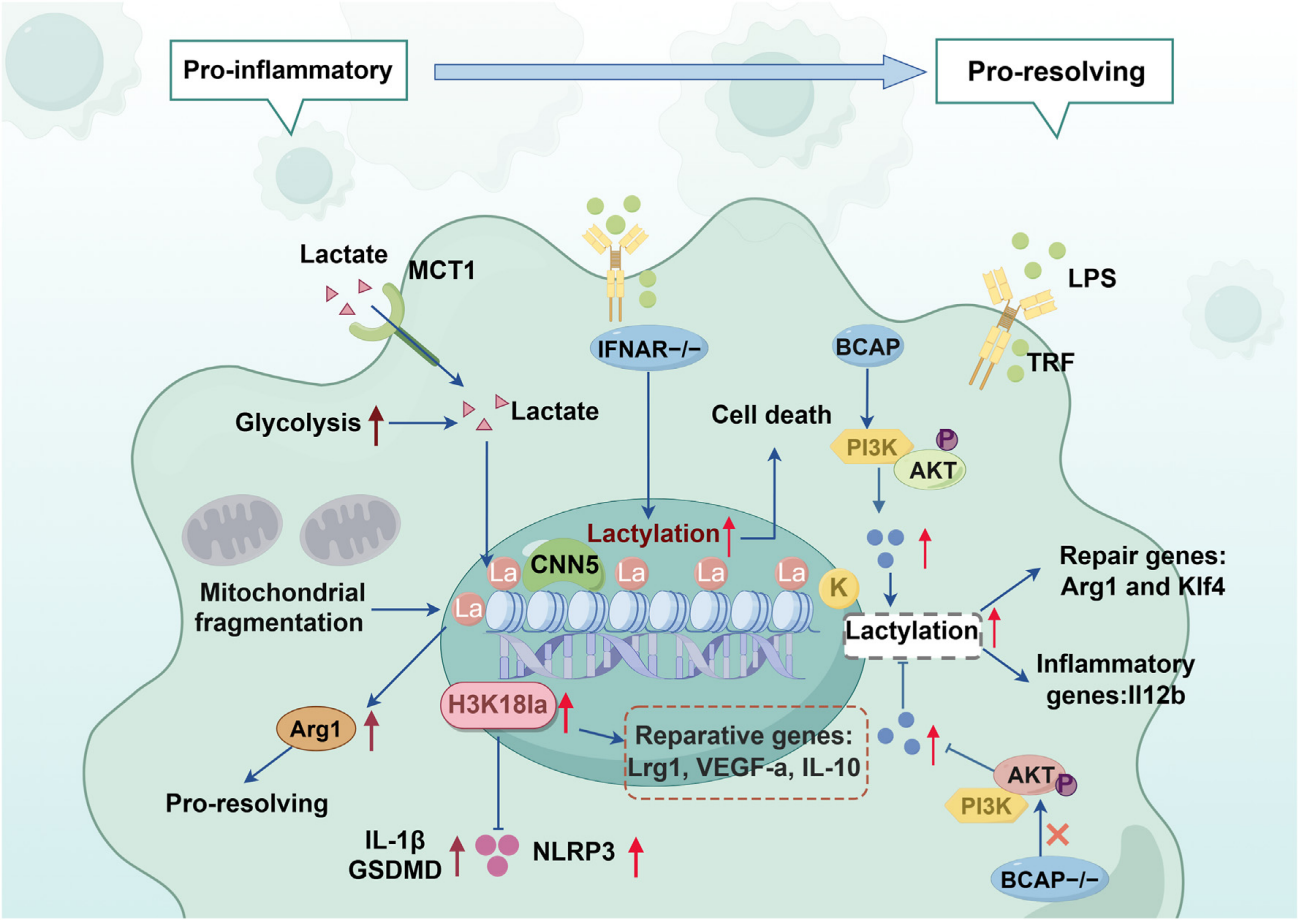
Role of histone lactylation changes in macrophages in Inflammatory related diseases. Histone lactate modification plays a crucial role in regulating the transformation of macrophage phenotypes, thereby maintaining the balance between inflammation and resolution in inflammatory diseases. In the presence of Nala or elevated histone lactylation levels, BCAP^−/−^ bone marrow-derived macrophages (BMDMs) were able to upregulate Arg1 expression to levels comparable to wild-type (WT) BMDMs, with a significant increase in Klf4 levels. During the early stages of myocardial infarction (MI), increased lactate levels in macrophages promote histone lactylation, which induces transcription of Lrg1, VEGF-a, and IL-10. Furthermore, high lactate concentrations in cells with fragmented mitochondria enhance histone lactylation, facilitating the transition of macrophages to an anti-inflammatory phenotype.

In recent years, studies have highlighted the significance of macrophage histone lactylation in regulating polarized phenotypes in various inflammatory diseases, including atherosclerosis, myocardial infarction, and colitis. A recent study revealed that MCT4 is highly expressed in macrophages during atherosclerosis, with MCT4 deficiency enhancing H3K18 lactylation (H3K18la) through the p300 acetyltransferase, thereby initiating the transcription of anti-inflammatory genes in macrophages. Notably, the p300 inhibitor C646 reversed the effects associated with elevated H3K18la.^113^ Furthermore, increased lactate efflux mediated by MCT4 in myocardial cells heightened H4K12 lactylation in macrophages, facilitating inflammatory infiltration in the microenvironment during diabetic cardiomyopathy.^114^ Collectively, these findings suggest that MCT4-mediated lactate accumulation is a significant contributor to abnormal histone lactylation levels in macrophages during inflammatory diseases, indicating that histone lactate modification plays a crucial role in the pathological progression of these conditions. Studies by Zhen et al.,^115^ and Sun et al.,^116^ utilizing a DSS-induced mouse model of ulcerative colitis, demonstrated significantly elevated histone lactylation levels (H3K18la) compared to control subjects. Notably, modulation of lactate levels through exogenous intervention resulted in corresponding changes in lactylation patterns, suggesting that alterations in macrophage H3K18 lactylation may be implicated in the pathogenic mechanisms underlying ulcerative colitis. During myocardial infarction (MI), monocyte-derived macrophages undergo rapid and substantial activation. In the early stages of MI, macrophages experience metabolic reprogramming, where elevated lactate promotes histone lactylation, inducing the transcription of genes associated with the monocyte repair response, such as Lrg1, Vegf-a, and IL-10. Additionally, IL-1β-dependent recruitment of GCN5 enhances H3K18la and the expression of repair genes. Conversely, inhibition of H3K18la increases serum inflammatory cytokine levels and post-MI cardiac inflammatory cell infiltration, leading to impaired angiogenesis, exacerbated cardiac remodeling, and diminished cardiac function.^117^ Clinical evidence underscores the relevance of histone lactylation in human disease. A recent cohort study of critically ill patients revealed distinct H3K18la patterns in peripheral blood samples compared to healthy controls. Notably, patients with higher H3K18la levels exhibited increased concentrations of both anti-inflammatory (IL-10) and pro-inflammatory (IL-6) cytokines, suggesting a complex regulatory role in inflammatory responses. The clinical significance of H3K18la is further emphasized by its positive correlation with established severity indices, including APACHE II scores and day 1 SOFA scores, as well as clinical outcomes such as ICU length of stay and duration of mechanical ventilation^118^ (Fig. 4).

The dysregulation of histone lactylation in inflammatory diseases is closely linked to metabolic shifts and changes in the cellular environment, which drive alterations in lactate metabolism and mitochondrial dynamics, particularly within macrophages. Elevated lactate levels resulting from aerobic glycolysis promote histone lactylation and alter gene expression, favoring anti-inflammatory or repair-associated responses. In this regulatory process, key enzymes such as MCT4 (which regulates lactate efflux) and p300 acetyltransferase play crucial roles in modifying specific histone sites like H3K18la. In summary, histone lactylation has emerged as a critical epigenetic modification with a dual role in promoting macrophage phenotypic transitions (e.g., from M1 to M2) while enhancing macrophage plasticity. This dual role allows macrophages to adapt flexibly to the demands of specific pathological microenvironments. However, the precise molecular mechanisms through which histone lactylation influences macrophage function in the pathogenesis of inflammatory diseases remain incompletely understood. Further investigations employing diverse experimental approaches are essential to elucidate the complex regulatory networks underpinning this epigenetic modification in inflammatory pathophysiology, particularly in determining how histone lactylation interacts with other epigenetic marks to collectively regulate macrophage plasticity and function.

### Muscle regeneration

Recent studies have increasingly highlighted the role of lactate-mediated histone lactate modification in various pathological processes. In the context of skeletal muscle injury and regeneration, evidence suggests that lactate acts not merely as a metabolic byproduct but as a key signaling molecule that orchestrates the regenerative response through epigenetic regulation.^119^ When skeletal muscle is injured, lactate promotes the differentiation of myogenic cells in vitro and activates muscle regeneration in vivo.^120^^,^^121^ Mechanistically, elevated exogenous lactate induces histone H3 lysine 9 lactylation (H3K9la), enhancing the transcription of neuraminidase 2 (Neu2), a positive regulator of myoblast differentiation.^119^ In an ischemic skeletal muscle injury model, increased lactate levels restore macrophage polarization and improve muscle reperfusion and regeneration.^121^ However, the specific mechanisms by which lactate regulates macrophage polarization and contributes to muscle regeneration after injury remain poorly understood. A recent study addressed this gap, revealing that in an ischemia-induced muscle injury model, histone lactylation levels in macrophages significantly increased over time. Notably, changes in H3K18la genome enrichment between days 2 and 4 post-injury predicted subsequent gene expression changes from days 4–7, indicating a transition from pro-inflammatory to pro-regenerative macrophage phenotypes. These findings underscore the importance of histone lactylation dynamics in macrophage function during muscle regeneration.^122^ This emerging field emphasizes the intricate relationship between cellular metabolism and epigenetic regulation in tissue repair, positioning histone lactylation as a novel mechanism coordinating the complex process of muscle regeneration. However, further investigation is needed to elucidate the upstream regulators and downstream effectors of histone lactylation-mediated signaling in macrophages within the injured muscle microenvironment.

### Fibrotic diseases

**Fig. 5:**
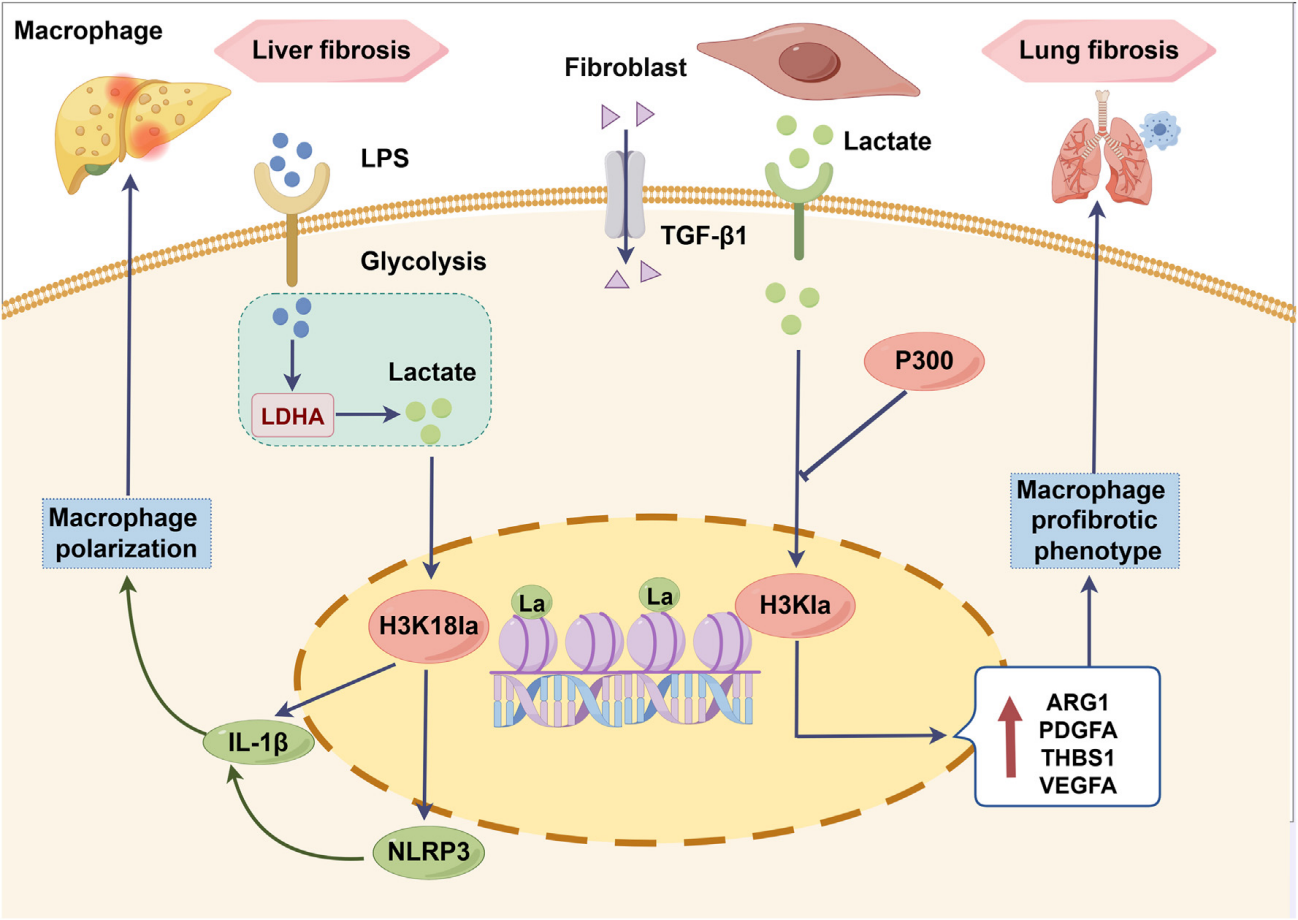
Role of histone lactylation changes in macrophages in fibrotic diseases.

Histone lactylation has recently emerged as a novel modification closely associated with various fibrotic diseases, including pulmonary fibrosis and liver injury.^117^^,^^123^^,^^124^ Previous research has demonstrated that lung fibroblasts exhibit enhanced aerobic glycolysis, which results in elevated lactate levels in the fibrotic lung microenvironment. This metabolic alteration influences epigenetic modifications and cellular behavior.^125^^,^^126^ In pulmonary fibrosis, studies have shown that lactate accumulation resulting from enhanced glycolysis promotes pro-fibrotic mediator expression in naive alveolar macrophages through histone lactylation. Specifically, increased histone lactylation has been observed at the proximal promoter regions of pro-fibrotic genes such as ARG1, PDGFA, THBS1, and VEGFA.^127^ Moreover, lactate derived from lung myofibroblast-conditioned media induces p300-mediated histone lactylation, reinforcing the pro-fibrotic phenotype in macrophages.^127^ Environmental factors, such as PM2.5 exposure, can initiate a pathogenic cascade by stimulating glycolysis and subsequent lactate production in macrophages, leading to elevated histone lactylation levels in fibrotic lung tissue.^128^ Similarly, a recent study using a CCl4-induced liver injury mouse model revealed that Kupffer cells exhibit increased glycolysis and histone lactylation during liver injury and fibrosis progression. Notably, treatment with Sal B and 2-DG significantly reduced glycolysis and inflammatory markers (PKM2, LDHA, NLRP3, and IL-1β) in isolated Kupffer cells, suggesting the therapeutic potential of targeting this pathway.^129^ Collectively, these findings underscore that enhanced glycolytic activity is a critical factor driving the increase in histone lactate levels (H3K18la). This positions H3K18la as a promising therapeutic target for modulating macrophage polarization in fibrotic diseases, offering new perspectives for anti-fibrotic interventions (Fig. 5).

Histone lactylation, a newly discovered modification, is closely associated with various fibrotic diseases, including pulmonary fibrosis and liver injury. Lactate promotes the expression of pro-fibrotic mediators in naïve alveolar macrophages, with significantly increased histone lactylation concentrations observed in the promoter regions of ARG1, PDGFA, THBS1, and VEGFA. Lactate produced during glycolysis can enhance the expression of NLRP3 and IL-1β by regulating histone lactylation in Kupffer cells.

### Excitatory and stress responses

All organisms are constantly exposed to various stressors, including environmental factors (such as temperature fluctuations and pathogens) and internal factors (such as cellular stress, mutations, and aging).^130^ Mammalian cells respond to these stresses and stimuli by activating mechanisms that support cellular function, thereby maintaining homeostasis within the microenvironment and the organism.^131^ During the stress response, cells undergo significant nuclear changes, including histone lactylation. Recent studies have underscored the importance of histone lactylation in stress-induced cellular responses. For instance, research on cold stress found that cold stimulation drives macrophage differentiation through histone lactylation induced by intracellular lactate accumulation. This modification activates STAT6 transcription and upregulates repair-associated genes, suggesting that histone lactylation is a key regulatory factor in balancing M1-like and M2-like macrophage populations during stress responses.^132^ Furthermore, studies using both in vitro neuronal excitation and electroconvulsive shock (ECS) models demonstrated that increased lactate levels enhance histone lactylation in mouse brain cells, including microglia. This finding establishes a novel link between neuronal activity and histone lactylation-mediated signaling in macrophages under pathological conditions.^133^ Collectively, these findings highlight the role of histone lactylation in connecting cellular metabolism to stress responses across various cell types and contexts. Future research should focus on elucidating the precise molecular mechanisms governing stress-induced histone lactylation, particularly exploring potential therapeutic applications in stress-related disorders and developing targeted strategies to modulate this epigenetic modification for therapeutic benefit.

### Histone lactylation levels are increased in clinical disease samples

Recent studies indicate that histone lactylation levels are elevated in various diseases, correlating closely with disease progression, severity, and poor prognosis (Supplementary Table S1). Evidence from multiple sources supports this association across different cancer types and disease states. For instance, Li et al. reported significantly elevated levels of both pan-lysine lactylation (Pan Kla) and H3K18 lactylation (H3K18la) in tumor tissues compared to adjacent non-cancerous tissues. Increased H3K18la levels positively correlated with advanced AJCC stages, suggesting its potential role as a key driver in pancreatic ductal adenocarcinoma (PDAC) pathogenesis and progression.^134^ Similarly, H3K18la levels are markedly elevated in endometriosis^135^ and septic shock,^118^ and they correlate significantly with the severity and prognosis of critically ill patients.^118^ The prognostic significance of histone lactylation is further exemplified in triple-negative breast cancer (TNBC), where H4K12 lactylation (H4K12la) shows markedly higher expression in tumor tissues. Follow-up data revealed a stark difference in median overall survival between patients with high versus low H4K12la expression (35 versus 49 months, respectively), highlighting its potential as a prognostic biomarker.^136^ In ocular melanoma, immunofluorescence analyses demonstrated significantly elevated levels of both H3K18la and Pan Kla compared to normal melanocyte tissues, indicating that elevated histone lactylation levels are associated with an unfavorable prognosis for these patients.^137^ Beyond its role in disease progression, histone lactylation has emerged as a critical regulator of treatment response,^138^ particularly in cases of chemotherapy resistance. Elevated H3K9 lactylation (H3K9la) levels correlate with temozolomide resistance in recurrent GBM.^138^ Additionally, colorectal cancer patients resistant to bevacizumab treatment show increased H3K18 lactylation levels, suggesting its role in modulating therapeutic efficacy.^139^ Collectively, this research underscores histone lactylation as a promising diagnostic and prognostic biomarker for various diseases.

Recent technological advances have significantly enhanced our ability to investigate histone lactylation in macrophages. Mass spectrometry-based proteomics has emerged as a powerful tool for identifying novel lactylation sites and quantitatively profiling lactylation dynamics in cells under different pathological and physiological conditions.^140^ Since their introduction around 2019, pan-lactyllysine antibodies have been developed and validated for Western blot and ChIP-seq analyses, shedding light on global lactylation patterns and genome-wide distribution.^17^ Recent studies have successfully utilized these antibodies for immunofluorescence microscopy, allowing tracking of lactylation changes at the single-cell level.^141^ Notably, the integration of lactylation analysis through CUT&Tag chromatin profiling with metabolomics and transcriptomics has unveiled intricate connections between cellular metabolism, histone modifications, and gene regulation.^124^ Despite these advancements, challenges remain in detecting site-specific lactylation events and distinguishing them from other acylation modifications. Future methodological developments, particularly in targeted mass spectrometry and modification-specific antibodies, will be crucial for fully elucidating the biological roles of histone lactylation in cellular function.

### Drugs targeting macrophage histone lactylation for disease treatment

Histone lactylation is crucial for regulating macrophage functions, prompting research into drugs that target this epigenetic modification as a novel approach to disease treatment. While many studies are ongoing, several inhibitors and compounds have demonstrated promise in inhibiting histone lactylation. Key agents include LDH inhibitors, glycolytic pathway inhibitors, MCT inhibitors, and natural compounds and their derivatives, as well as HDAC inhibitors.

In disease management, LDH inhibitors play a significant role in modulating histone lactylation. Research has shown that oxamate and dichloroacetate (DCA) can lower intracellular lactate levels, inhibiting H3K18 lactylation and subsequently reducing the expression of genes linked to hepatic stellate cell (HSC)^124^ activation. Furthermore, oxamate can inhibit lactate production in AGEs-induced renal tubular epithelial cells, diminishing H3K14 lactylation at the Klf5 promoter. This action attenuates the epithelial-to-mesenchymal transition (EMT) and slows the progression of diabetic nephropathy.^142^ Another LDHA-specific inhibitor, (R)-GNE-140, similarly reduces intracellular lactate, decreasing the repression of Neu2 expression dependent on H3K9 lactylation, thereby impacting myogenesis.^119^ Additionally, the combination of oxamate and CAR-T therapy reduces H3K18 lactylation, inhibits lactate production, and downregulates the activity of CD39, CD73, and CCR8 gene promoters. This alters the immunosuppressive tumor microenvironment (TME) and promotes immune activation.^103^ Collectively, these findings underscore the therapeutic potential of LDH inhibitors in modulating histone lactylation across various pathological contexts.

Pharmacological inhibition of glycolysis can also lower histone lactylation levels, thereby decelerating disease progression. Researchers found that 2-deoxy-D-glucose (2-DG) treatment reduces H3K18 lactylation, leading to decreased liver fibrosis.^143^ Similarly, 2-DG diminishes H3K9 lactylation in endothelial cells, weakening pathological angiogenesis.^144^ In preclinical models resistant to bevacizumab, the combination of 2-DG and bevacizumab exhibited significant therapeutic efficacy, suggesting a novel strategy to enhance bevacizumab's clinical effectiveness in colorectal cancer by inhibiting histone lactylation.^139^ Moreover, pharmacological inhibition of MCT4 can mitigate disease progression through histone lactylation modulation. Studies indicate that the MCT4 inhibitor VB124 significantly slows atherosclerosis progression in apoe-deficient mice fed a high-fat diet by enhancing p300-mediated H3K18 lactylation, indicating its potential clinical application in atherosclerosis^113^ treatment. Additionally, VB124 has been shown to protect cardiac function in type 2 diabetic mice, likely by modulating histone lactylation in macrophages to exert anti-inflammatory effects.^114^

Various natural compounds and their derivatives also exhibit therapeutic effects against multiple diseases by regulating histone lactylation. For instance, in CCl4-induced liver injury mouse models, salvianolic acid B (Sal B) has been shown to reduce histone lactylation levels in macrophages, thereby inhibiting M1 polarization of Kupffer cells and ultimately alleviating liver damage.^129^ In castration-resistant prostate cancer (CRPC) cells, evodiamine inhibits the expression of HIF1α and H3K18la, effectively blocking lactate-induced angiogenesis. This discovery suggests a potential new therapeutic avenue for prostate cancer treatment targeting histone lactylation modifications.^145^ Similarly, both royal jelly acid and demethylzeylasteral (DML) have demonstrated inhibitory effects on hepatocellular carcinoma by suppressing histone lactylation, indicating that modulation of histone lactylation^146^^,^^147^ could be a promising strategy for treating this cancer type. Furthermore, stiripentol and 20(S)-ginsenoside Rh2 have been reported to enhance glioblastoma sensitivity to temozolomide^138^ and improve all-trans retinoic acid (ATRA) resistance in acute promyelocytic leukemia (APL),^148^ respectively, providing potential therapeutic strategies to overcome tumor resistance. Combination therapy is a widely employed approach in cancer treatment. Recent studies indicate that a combination therapy involving PI3K/MEK/PORC inhibitors restores lactate-mediated H3K18la in tumor-associated macrophages (TAMs) while suppressing macrophage phagocytosis, thereby promoting prostate cancer^149^ progression. Notably, these natural compounds often exhibit multi-target effects, suggesting that their inhibition of histone lactylation may be indirect and warranting further investigation into their specific mechanisms.

In the evolving landscape of cancer research, an increasing number of small-molecule inhibitors targeting histone deacetylases (HDACs) have been developed, demonstrating efficacy in delaying tumorigenesis. Notable examples include vorinostat (SAHA), belinostat, and panobinostat.^150^ Recent studies have revealed that the molecular mechanisms of HDAC inhibitors (HDACi) in disease treatment are linked to the regulation of histone lactylation. Specifically, the I-class HDAC inhibitors apicidin (A8176) and MS275 (ApexBio) suppress the expression of genes that promote hepatic stellate cell (HSC) activation by inducing H3K18ac, which competitively interferes with H3K18la. These findings underscore the complexity of histone modification dynamics within cells and suggest that I-class HDAC inhibitors may be pivotal in regulating histone lactylation, offering new therapeutic strategies for related diseases.^124^ Additionally, in studies on pancreatic ductal adenocarcinoma (PDAC), the HDAC inhibitor TSA significantly elevated histone lactylation levels, thereby delaying PDAC growth and progression.^134^ Conversely, research indicates that TSA treatment can induce histone acetylation, inhibiting the proliferation, migration, and invasion of head and neck squamous cell carcinoma (HNSCC) cells.^151^ Since both lactylation and acetylation are regulated by HDAC1 and HDAC3, these modifications may compete within cells, influencing gene expression and cellular functions. Thus, current HDAC inhibitors targeting histone lactylation often lack specificity. Future research should prioritize the development of inhibitors that selectively target specific HDAC isoforms to delineate the distinct functions of lactylation and acetylation. Furthermore, integrating genomic and proteomic approaches to analyze these modifications comprehensively will reveal their dynamic changes across different cellular states, providing critical insights for the application of HDAC inhibitors in disease treatment.

## Conclusion

Collective evidence indicates that alterations in the differentiation, polarization, re-polarization, and activation of macrophage populations in the local environment significantly influence various autoimmune diseases, inflammatory disorders, neurological conditions, and diverse tumors.^2^^,^^152^ Since the seminal discovery of histone lactylation modifications in bone marrow-derived macrophages by Zhang et al., in 2019, this emerging epigenetic modification has attracted increasing attention.^17^ This review systematically summarizes the current understanding of the pathological triggers and molecular mechanisms that lead to altered histone lactylation levels in disease states. We explore how macrophages leverage lactate-induced histone lactylation to regulate downstream gene expression programs that orchestrate various pathological processes, including the initiation and resolution of inflammation, tumor development and metastasis, drug resistance, and fibrosis promotion and regression.

We identify several critical knowledge gaps in the field of histone lactylation: (1) the complete enzymatic machinery involved in histone lactylation, including its writers, readers, and erasers, remains largely undefined; (2) the intricate interplay between lactylation and other histone modifications requires further exploration; and (3) the site-specific functions and downstream signaling pathways associated with lactylation need systematic characterization. To address these challenges, we propose an integrated approach that combines cutting-edge genomic techniques with advanced technologies to comprehensively profile histone lactylation landscapes in macrophages, track their temporal dynamics, and elucidate their roles in disease pathogenesis. Notably, emerging clinical evidence indicates that levels of H3K18 lactylation in peripheral blood mononuclear macrophages correlate with disease severity across multiple conditions, including cancers, underscoring its potential as a novel biomarker. However, there is an urgent need for large-scale, multicenter clinical studies to validate the prognostic significance of macrophage histone lactylation signatures. This review synthesizes current knowledge while providing a forward-looking perspective on leveraging histone lactylation as a therapeutic target, acknowledging the substantial challenges in translating these fundamental insights into clinical applications. Thus, substantial efforts are still required to bridge the gap between histone lactylation levels in macrophages and their clinical significance.

## Outstanding questions

We recommend combining genomic and advanced technologies for in-depth analyses of histone lactylation modifications and the key enzymes involved in macrophage histone modification. This research should track the fate of these modifications and determine their functions in disease pathology. Given the dual role of histone lactylation in macrophage polarization, it is essential to explore how it selectively modulates macrophage plasticity in different disease contexts. Understanding the distinct functional roles of lactylation and acetylation, both regulated by HDAC1 and HDAC3, is crucial, especially because current HDAC inhibitors targeting histone lactylation often lack specificity.

Additionally, large-scale, multicenter clinical studies are essential for establishing robust correlations between histone lactylation patterns in peripheral blood mononuclear macrophages and clinical outcomes, including disease severity and prognosis. This evidence base is critical for validating histone lactylation as a therapeutic target.

Furthermore, creating comprehensive lactylation maps through proteomics and modificationomics across various systems—particularly in human and mouse macrophages—will enhance our understanding of the crosstalk between histone lactylation and other post-translational modifications, thereby elucidating macrophage polarization and function in various diseases.

**Search strategy and selection criteria**

Data for this review were identified through comprehensive searches in PubMed and MEDLINE. The search terms used included combinations of keywords related to histone lactylation, macrophage activation, and their roles in various diseases. Specific search terms included: “histone lactylation” OR “lactylation” OR “histone lysine lactylation” OR “epigenetic regulation” OR “macrophage activation” OR “macrophage polarization” OR “lactate and macrophages” OR “immune response and histone lactylation” OR “macrophage plasticity and epigenetics” OR “tumor-associated macrophages” OR “microglia and histone modification” OR “inflammation and histone modification” OR “lactate metabolism and macrophages” OR “tumor progression and histone lactylation” OR “immune cells and epigenetic regulation” OR “neuroinflammation and epigenetics” OR “chronic diseases and lactylation” OR “histone modifications in cancer”. Articles published in English from 2010 to 2024 were included. Selected studies were individually examined to determine their eligibility for inclusion in the review. Each article was meticulously evaluated to ensure a comprehensive overview of the topic.

## Contributors

C. He, C. Bao, and J. Huang conceived and designed the study. C. Bao, X. Ying, and F. Wang drafted of the main text. Y. Hou, D. Wang, L. Zhu prepared the figures and tables. C. Bao, Q. Ma, J. Huang and C. He revised figures/tables and manuscript. All authors read and approved the final version of the manuscript, and ensure it is the case.

## Declaration of interests

The authors have no conflicts of interest to declare.
