# Supplementary material for: Histone lactylation in macrophage biology and disease: from plasticity regulation to therapeutic implications
Source: eBioMedicine. 2024 Dec 10;111:105502. doi: 10.1016/j.ebiom.2024.105502 (PMC11697715; doi:10.1016/j.ebiom.2024.105502)
Supplement: Supplementary Table S1 [file mmc1.docx]

**Table1. Histone lactylation levels are increased in clinical disease samples**

| Diseases | Samples | | | Histone lactylation levels | | Pathological mechanism | | References |
| --- | --- | --- | --- | --- | --- | --- | --- | --- |
| PDAC | PDAC (n=74) and healthy controls (n=72) | | | H3K18la and pan Kla | | The increased H3K18la levels were positively correlated with more advanced AJCC stages | | ^134^ |
| Endometriosis | Endometriosis patients (n=20) and control samples (n=20) | | | H3K18la | | H3K18Ia may have a role in the etiology of the endometriosis | | ^135^ |
| Septic shock | Shock patients（n=24）and healthy volunteers (n=13) | | | H3K18la | | H3K18la correlates significantly with the severity and prognosis of critically ill patients. | | ^118^ |
| TNBC  Ocular melanoma  GBM | Patients with TNBC (n=112) and adjacent non-cancer controls (n=84)  Ocular melanoma tissues (n=82) and normal melanocyte tissues (n=28)  Recurrent GBM tissues (n=18) and primary GBM tissues (n=18) | | | H4K12la  H3K18la and Pan Kla  H3K9Ia | | H4K12la expression correlated positively with Ki-67 and inversely with overall survival in TNBC  Elevated histone lactylation levels are associated with unfavorable prognosis for patients with ocular melanoma  H3K9Ia accumulation is closely related to TMZ resistance in GBM | ^136^  ^137^  ^138^ | |
| CRC | | Stage I-III CRC patients (n=10) and adjacent normal tissues (n=10) | H3K18la | | Increased H3K18la levels confer resistance to bevacizumab and were associated with poor survival for patients with CRC. | | ^139^ | |

PADC: pancreatic ductal adenocarcinoma; TNBC: triple-negative breast cancer; CRC: colorectal cancer; OSCC: oral squamous cell carcinoma; GBM: glioblastoma;
